# Alpha oscillatory activity reveals focused-attentional disparity between cochlear implant users and normal hearing listeners

**DOI:** 10.1038/s41598-026-52434-6

**Published:** 2026-05-09

**Authors:** Brilliant, Irina Schierholz, Pascale Sandmann, Andrej Kral

**Affiliations:** 1 Department of Experimental Otology, Hannover Medical School (MHH) at Lower Saxony Center for Biomedical Engineering, Implant Research and Development (NIFE), Joint Research Institute of AudioNeuro Technology (VIANNA), Stadtfelddamm 34, 30625 Hannover, Germany; 2 Cluster of Excellence “Hearing4all”, Oldenburg, Germany; 3 Department of Otorhinolaryngology, Head and Neck Surgery, Faculty of Medicine, University Hospital Cologne, Cologne, Germany; 4 Department of Otolaryngology, Head and Neck Surgery, Carl Von Ossietzky University Oldenburg, Oldenburg, Germany

**Keywords:** Neuroscience, Psychology, Psychology

## Abstract

Cochlear implants (CIs) allow hearing-impaired subjects to regain access to speech. However, the individual outcome variability remains high and unexplained. One contributing factor to the CI performance could be related to higher cognitive functions, which are not as well studied as in normal hearing (NH) populations. To study the cognitive processing in CI-users, in particular attentional processing, we compared the neural correlates of attention of a large cohort of CI-users (n = 37) to those of an age-matched group of NH-listeners (n = 37). The cognitive paradigm used here is a three-stimulus oddball paradigm in both passive and active listening conditions to isolate the attentional difference in unattended and attended task conditions. The addition of a third stimulus-type allowed us to specifically disentangling the attentional processing of task-relevant from the task-irrelevant stimuli. We expanded the previous ERP study by inspecting oscillatory activities. We localized the responsible brain areas and estimated local neural activities in three cortical regions: temporal transverse gyrus dorsolateral prefrontal cortex and inferior parietal lobule. We found alpha activity (8–12 Hz) to be the most relevant correlate of top-down auditory attention. The result on alpha activity suggests that CI-users may have directed their attention toward task-irrelevant stimuli, thereby impeding their focus on task-relevant stimuli. In sum, this study provides important insight into the cognitive attentional processing in CI-users; those may contribute in high listening effort and CI performance variability.

**Supplementary Information:**

The online version contains supplementary material available at 10.1038/s41598-026-52434-6.

## Introduction

Cochlear implants (CIs) often are the only option to treat severe-to-profound hearing loss. Despite its success, CI performance shows a remaining unexplained variability^1^. Electric hearing through a CI differs in many aspects from acoustic hearing and does not provide all distinctive cues, for example for speech understanding^2–4^. Hearing with a CI, therefore, may involve more cognitive processing and require more attentional resources, thus resulting in higher listening effort^5,6^. Cognitive functions in CI-users are often evaluated using subjective behavioral measures^7^, however with mixed results^8^. Moreover, such assessments do not allow identification of the underlying neural sources^9^. Knowledge about the neural cognitive processes in CI-users would therefore provide important insights for understanding effortful listening in general and for better rehabilitation of CI-users in particular.

Through electroencephalography (EEG), brain activity related to auditory cognition can be objectively assessed^10^. Combined with paradigms such as the oddball task, which is a gold-standard in cognitive research, EEG allows the assessment of cognitive processes such as perceptual discrimination and attention^11^. The time-domain event-related potentials (ERPs) can be utilized as objective measures of perceptual- and cognitive processing. Early ERP components include the N1, reflecting activation in the auditory cortex and stimulus detection^12^; the P2, generated mainly in primary and secondary auditory cortex for processing of stimulus physical characteristics^13^; and the N2, indexing the early stimulus discrimination and attentional modulation^14,15^. The later P3 component reflects the higher-level cognitive processes^16,17^. In classic two-stimulus oddball task, the P3 has been shown to reveal larger amplitudes for infrequent target compared to frequent standard stimuli and to align with behavioral response latencies, confirming its role in higher-order processing^18^. By adding a third stimulus-type in the oddball paradigm: the novel stimulus, differentiation between two distinct P3-subcomponents is possible: Novelty-P3 (P3a for involuntary attention) and Target-P3 (P3b; task-relevant evaluation)^19–21^. Yet, Novelty-P3 has been further subdivided into an early and a late sub-subcomponents (early and late Novelty-P3)^22^, indicating the complexity of the underlying processes behind a single P3 component. In CI-users, ERP studies reliably showed reduced amplitudes and prolonged latencies for early ERP components (e.g. N1, P2 and N2) compared to normal hearing (NH) individuals^23,24^. However, P3 differences between CI-users and NH-listeners are less consistent^5,25–27^, possibly due to time-domain averaging, eliminating non-phase locked activity that often emerges at later latencies^28^.

A different approach, namely the time–frequency analysis technique captures the non-phase locked activity due to power averaging instead of amplitude. The resulting oscillatory responses have been used as neural signatures of various cognitive processes^29,30^, such as attention^31^ and memory^32^. Attention as a subdomain of cognition has been in fact closely linked to alpha oscillatory activity^33–35^. For example, during a selective attention task, alpha power decreases at electrodes over the contralateral hemisphere of the target location and consequently increases over the contralateral hemisphere of the distractor^36–38^. This has given rise to an inhibitory role of alpha oscillation, namely as mechanism to inhibit neuronal excitability of specific brain areas to suppress the processing of the distractor^39–41^. It remains debatable, whether this inhibitory role of alpha is an independent mechanism or whether it is an indirect mechanism of goal-directed target processing^42–44^. Nevertheless, the previous research on alpha oscillations and attention have been heavily focused on the selective attention paradigm, where distractor and target are presented simultaneously. In the auditory domain, increased alpha has also been linked to increased listening effort during noisy conditions, where the attended signal and the noise were presented simultaneously^45–47^, especially for cohort with hearing impairment^48^. It remains unclear whether CI-users might also already exhibit differences compared to NH-listeners in other types of attention beside selective attention (e.g. focused or focal attention)^49–52^.

In this study, we aim to identify the oscillatory signature of listening with CI in relation to attentional process. We analyzed in time–frequency manner the dataset, which was collected in conjunction with those from the previous ERP study^26^. We were interested in oscillatory markers of bottom-up and top-down processes^53^, where theta power may serve as a signature of bottom-up perceptual processing^54,55^ and alpha power as signature of top-down attention^56^. For that, a three-stimulus oddball paradigm, incorporating the typical frequent standard and the infrequent task-relevant target but also the infrequent but unique task-irrelevant novel stimulus. We hypothesize that due to perception uncertainty, it might be more challenging for CI-users to perceptually discriminate the task-relevant target from the standard, reflected in theta activity. Furthermore, we examine, whether the CI-users may experience difficulties in focusing their attention, namely being more distracted by the task-irrelevant novel stimulus^57^, which is hypothesized to be reflected in the alpha oscillatory activity. Finally, we estimate and investigate the neural responses to the three stimuli at three different cortical regions: the temporal transverse gyrus (TTG), the dorsolateral prefrontal cortex (DLPFC) and the inferior parietal lobules (IPL). These three regions were chosen, as each of them represents the three important brain networks in this task: the auditory network, the central executive network and the attentional network. In sum, the goal in this study is to examine a potential attentional difference between NH-listeners and CI-users, that may emerge during a simple auditory task and may contribute to the increased listening effort or variable performances observed in CI-users.

## Results

### ERP and TFRs: reduced neural responses for standard stimuli and in passive condition

**Fig. 1:**
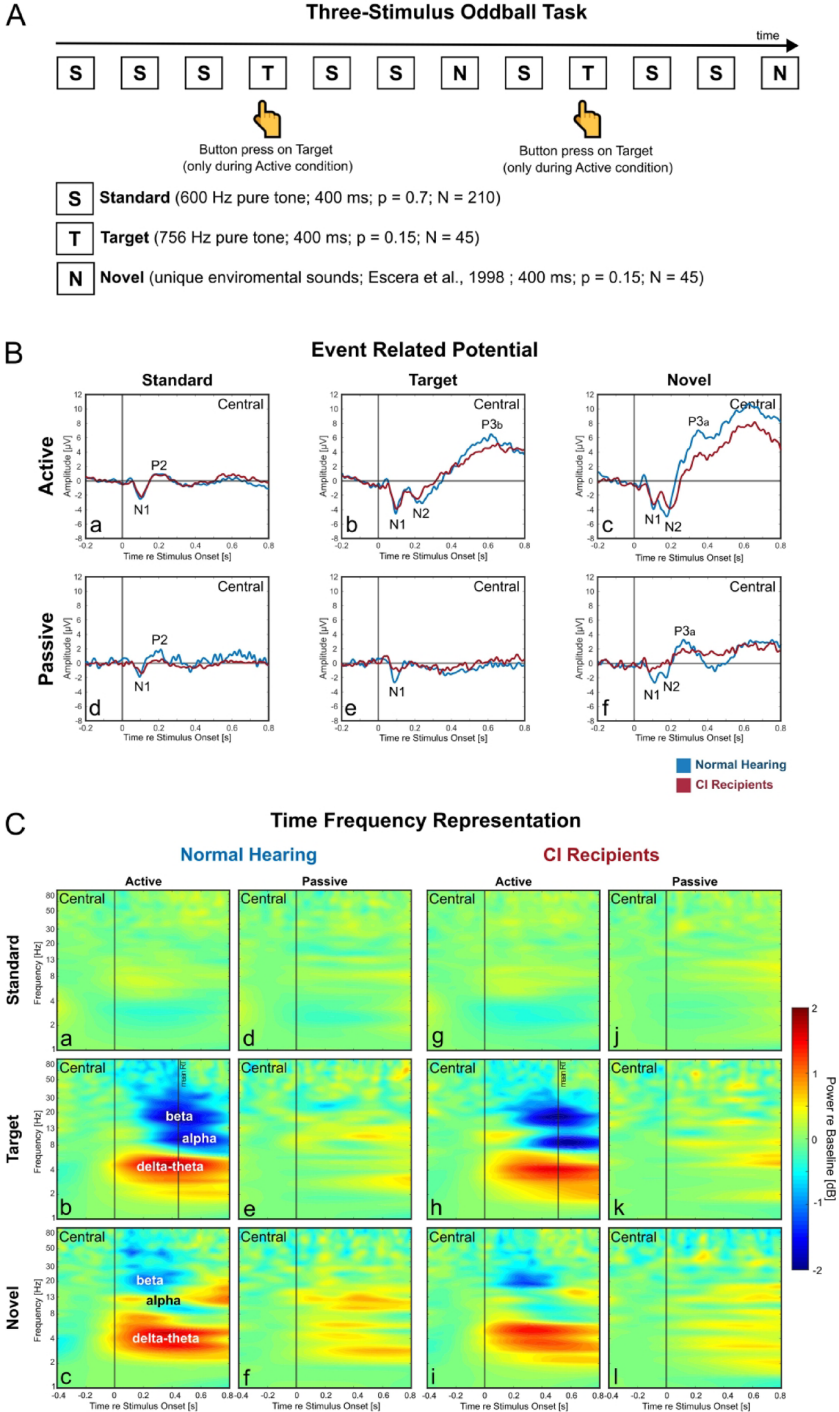
Three-Stimulus Oddball Paradigm: Experimental Design, Event-Related Potentials (ERPs) and Time–Frequency Representation (TRF). Experimental design of the Three-Stimulus oddball task (**A**), grand-mean ERPs (**B**) and grand-mean TFRs (**C**) of normal hearing (NH) and CI-users (CI) for all stimuli in both the active and passive condition for the Central ROI.

The three-stimulus oddball paradigm consisted of three stimulus-types: standard, target and novel stimuli and were performed in an active (button press on target) and in a passive condition (Fig. 1A). The central-ROI event-related potentials exhibited robust early ERP components (N1 and N2) for both groups, especially in the active condition (Fig. 1B a, b, c). In this condition, additional late ERP components (P3a, P3b) were also pronounced for both groups for target and novel stimuli (Fig. 1B b, c). In the passive condition, while N1 component were still observable, there were less appearance of late ERP components for both groups (Fig. 1B d, e, f). In sum, although the stimulus duration in this study is longer than the one used previously (400 ms instead of 200 ms), the observed ERP components in this study are comparable with result from the previous investigation^26^.

For both groups, the time–frequency representations (Fig. 1C) revealed pronounced oscillatory activities mainly in the active condition (Fig. 1C first and third columns) but less observable in the passive condition (Fig. 1C second and fourth columns). The oscillatory activities were also mainly pronounced for target and novel stimuli (Fig. 1C second and third rows) but less observable for standard stimuli (Fig. 1C first row). Target stimuli in the active condition (Fig. 1C b, h) elicited delta-theta synchronization, alpha desynchronization and beta desynchronization. Novel stimuli in the active condition (Fig. 1C c, i) elicited delta-theta synchronization, minor alpha synchronization followed by beta desynchronization. Target and novel stimuli during the passive condition (Fig. 1C e, f, k, l) had less clear oscillatory activities, where only minor synchronizations were observable.

### Active vs. passive: oscillatory activities as markers of active attention

**Fig. 2:**
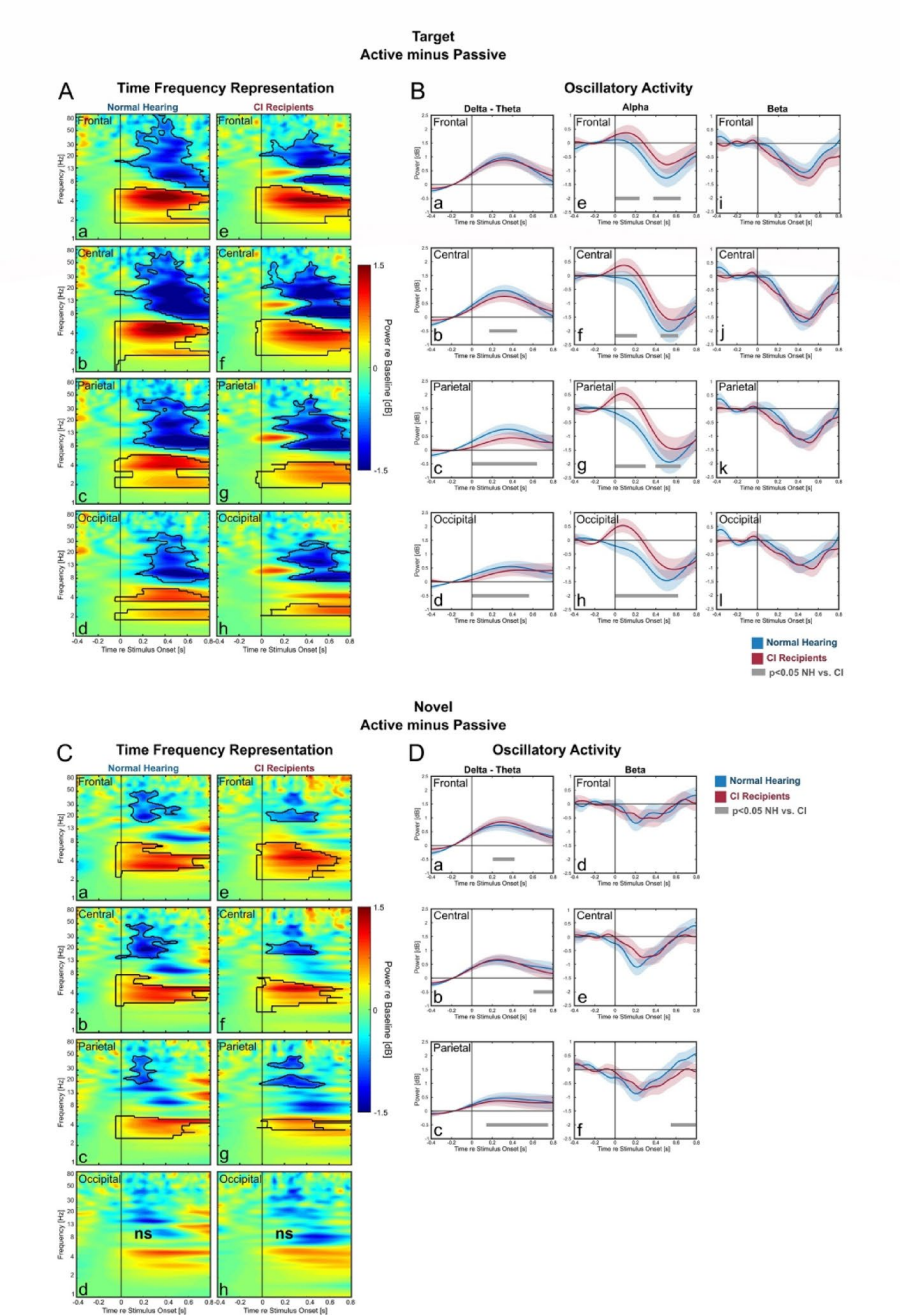
Comparing oscillatory activities in active vs. passive condition. Difference-TFRs between the active and the passive condition with the time-course of the oscillatory activities for target (**A**, **B**) and novel stimuli (**C**, **D**). The black contours in TFR plots denote significant differences between the two conditions (cluster-based permutation test, p < 0.05). The time-course of the oscillatory activities were only further plotted if they were significant in the TFR for at least one of the two group. In these oscillatory activity plots (right) the blue lines represent the frequency-bands average power for the NH-listeners, while red lines represent the average power for the CI-users (blue and red areas represent the SEM). Grey lines denote significant time-point differences between the two groups in a 150 ms sliding window (Bonferroni corrected independent samples t-test, p < 0.05).

To identify which oscillatory activities played a role in the attended vs. unattended conditions, we subtracted the TFRs in the passive from that in the active conditions. During target processing (Fig. 2A) we observed delta-theta amplification, followed by a reduction alpha-, and beta band in difference-TFR plots. These effects were observable in all ROIs and in both the NH-listeners and the CI-users. Next, we calculated the time-course of these delta-theta, the alpha and the beta effects and compared them between the groups (Fig. 2B). The CI-users exhibited less delta-theta amplification (Fig. 2B b-d) and less alpha reduction (Fig. 2B e–h) during target processing compared to NH-listeners. Beta reduction was, however, comparable between the two groups.

The analysis of the novel processing in active vs. passive comparison (Fig. 2C) revealed these oscillatory effects: a delta-theta amplification followed by a beta reduction, which were observable in all ROIs except the occipital ROI. The delta-theta amplification was slightly but significantly stronger in CI-users in the frontal region (Fig. 2D a, b) but became weaker in the parietal region (Fig. 2D c).

### Target vs. standard: comparable neural responses between CI-users and NH-listeners

**Fig. 3:**
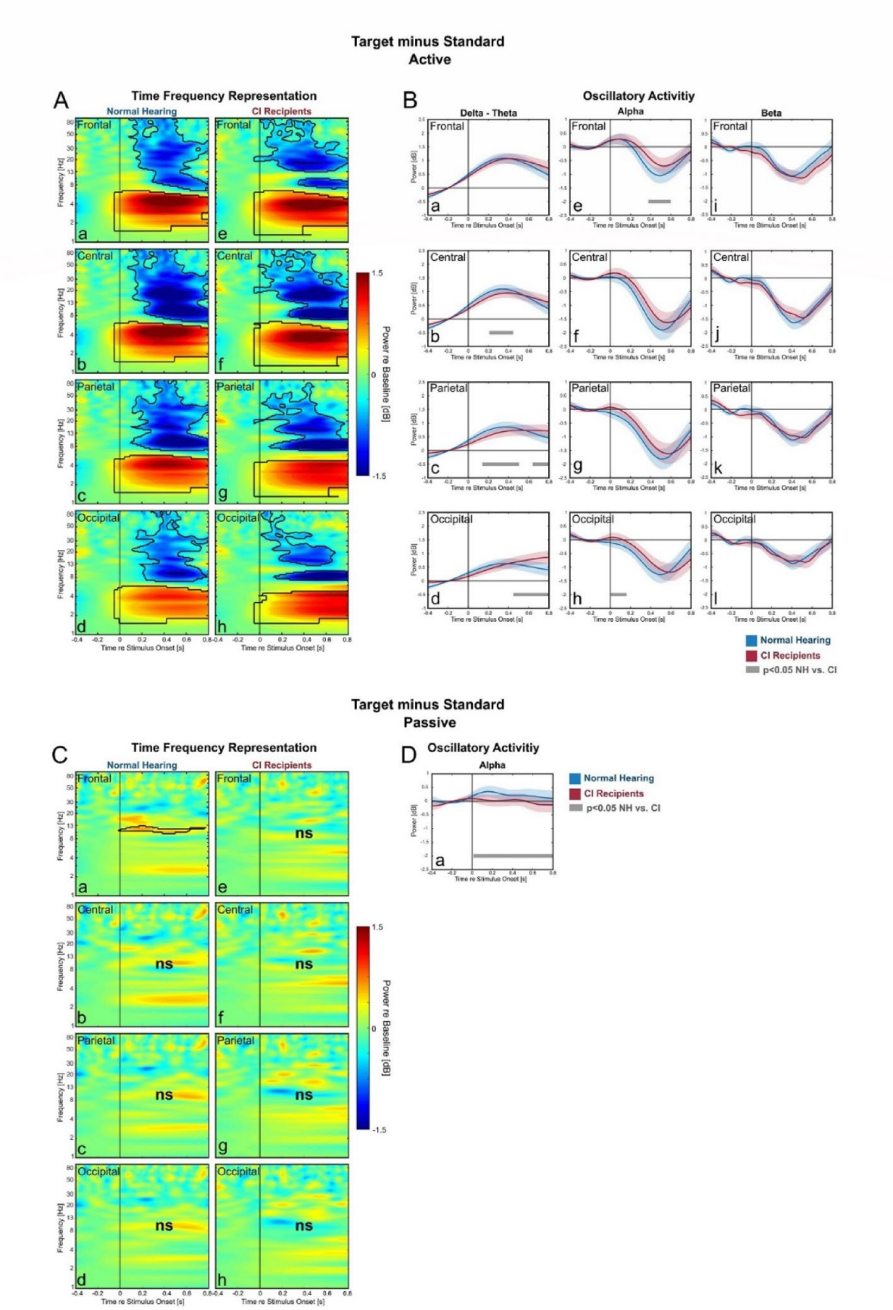
Comparing oscillatory activities between Target and Standard. Difference-TFRs between Target and Standard with the time-course of the oscillatory activities in the active (**A**, **B**) and the passive (**C**, **D**) conditions. In TFR plots (left): black contours denote significant differences between the two stimuli (cluster-based permutation test, p < 0.05, ns means there are no significant time–frequency regions found for the corresponding TFR). The time-course of the oscillatory activities were only further plotted if they were significant in the TFR for at least one of the two group. In these oscillatory activity plots (right): blue lines represent the frequency-bands average power for the normal hearing, while red lines represent this average power for the CI-users (blue and red areas represent the SEM). Grey lines denote significant time-point differences between the two groups (Bonferroni corrected independent samples t-test, *p* < 0.05, only significance above 150 ms was considered*).*

Comparing the deviating stimulus (task-relevant target or task-irrelevant novel stimuli) with the standard stimuli allows us to determine the oscillatory activities related to discrimination and processing of infrequent stimuli (compare^58,59^). Here, we compared both target vs. standard (Fig. 3) and novel vs. standard (see next section and Fig. 4) in both the active and the passive conditions.

During the active condition, the significant differences in target vs. standard comparison were found in delta-theta amplification and alpha–beta reduction, which were observable in all ROIs for both groups (Fig. 3A). However, in the active condition, the significant groups’ difference was only found in delta-theta band, while the effects in alpha and beta bands were overall not systematically different between the groups (Fig. 3B). In the passive condition, the only observed effect was the alpha amplification in NH-listeners (Fig. 3C). This was also further confirmed in the analysis of time-course (Fig. 3D).

### Novel vs. standard: stronger alpha reduction for CI-users

The comparison of novel vs. standard stimuli (Fig. 4A) gives insight into the processing of task-irrelevant stimuli. This comparison in the active condition revealed significant amplification delta-theta band and a reduction alpha–beta band. Similar to the previous target vs. standard comparison, delta-theta amplification in novel vs. standard comparison was also greater in NH compared to CI-users in all ROIs (Fig. 4B a-d). However, in this current comparison of novel vs. standard, CI-users exhibited a greater degree of alpha reduction in parietal and occipital ROIs compared to NH (Fig. 4B g, h).

During the passive condition, the comparison of novel vs. standard stimuli showed significant effects of delta-theta amplification and alpha amplification (Fig. 4C). Similar to the previous target vs. standard comparison, the alpha amplification only occurred in NH-listeners (Fig. 4C a, b) and not in CI-users. However, the delta-theta amplification in the current novel vs. target comparison could be now detected in both groups (Fig. 4C a, b, c, e, f, g, h). The degree of both alpha and delta-theta amplifications was significantly greater in NH-listeners compared to CI, as indicated by the time-course of delta-theta- and alpha amplification (Fig. 4D).

**Figure:**
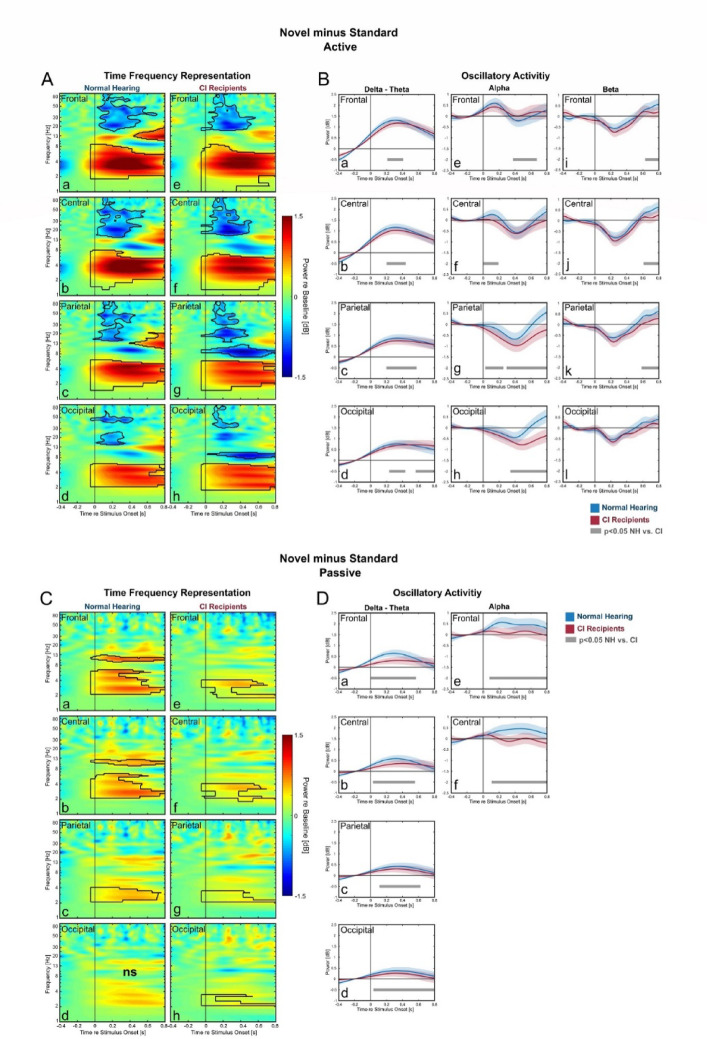
Comparison of oscillatory activities for Novel vs. Standard. Differences in TFRs between Novels and Standards with the time-course of the oscillatory activities during active- (**A**, **B**) and passive (**C**, **D**) condition. In TFR plots (left): black contours denote significant differences between the two stimuli (cluster-based permutation test, *p* < 0.05, ns means there is no significant time–frequency regions found for the corresponding TFR). The time-course of the oscillatory activities were only further plotted if they were significant in the TFR for at least one of the two group. In these oscillatory activity plots (right): blue lines represent the frequency-bands average power for the NH, while red lines represent this average power for the CI-users (blue- and red areas represent the SEM). Grey lines denote significant time-point differences between the two groups (Bonferroni corrected independent samples t-test, p < 0.05, only significance above 150 ms was considered).

### Target vs. novel: stronger alpha reduction for NH-listeners

**Fig. 5:**
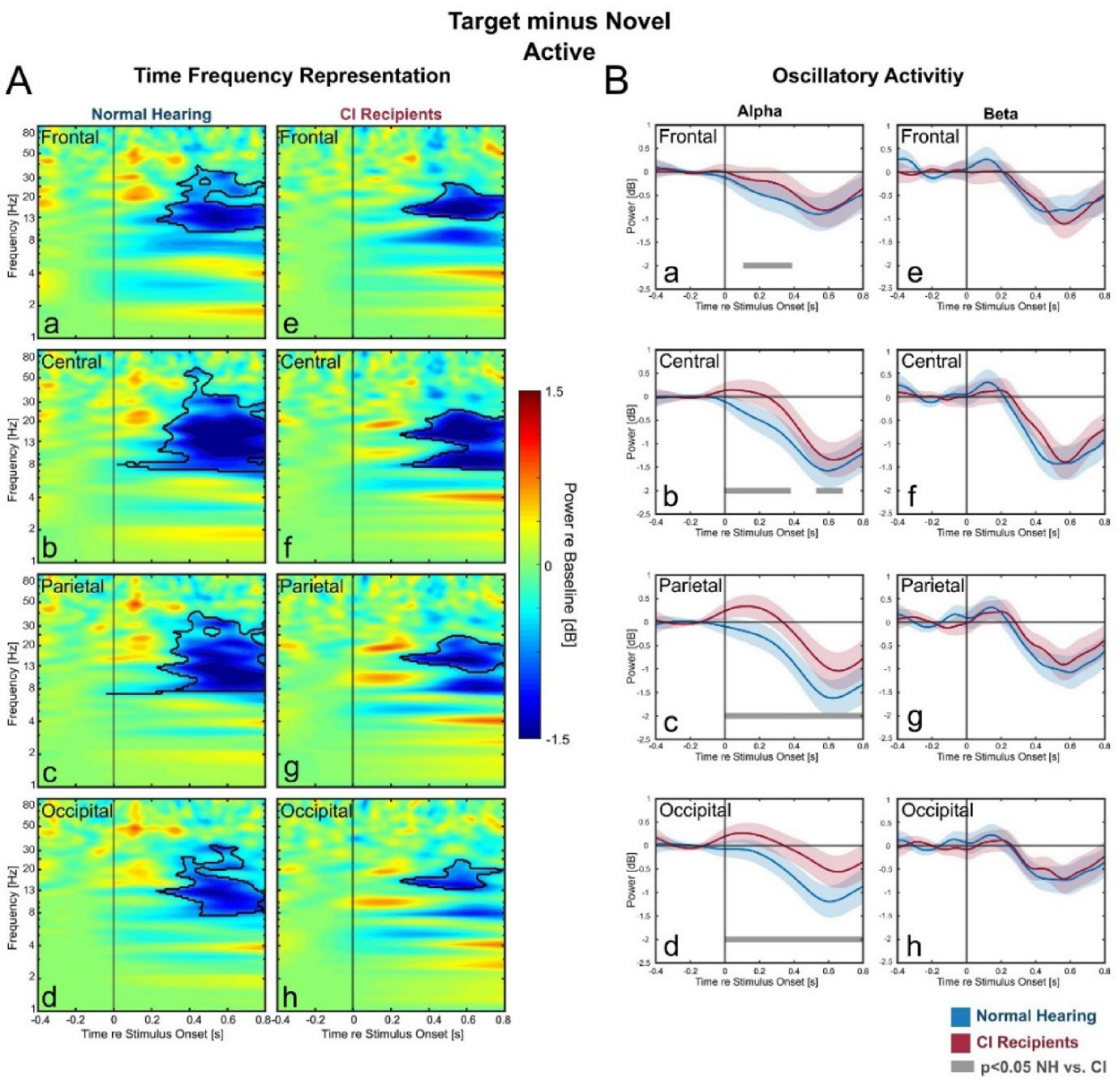
Comparing oscillatory activities for Novel vs. Standard. Difference-TFRs (left) between Target and Novel (**A**) with the time-course of the oscillatory activities (**B**) during active condition. In TFR plots (left): black contours denote significant differences between the two stimuli (cluster-based permutation test, *p* < 0.05). The time-course of the oscillatory activities were only further plotted if they were significant in the TFR for at least one of the two group. In these oscillatory activity plots (right): blue lines represent the frequency-bands average power of the normal hearing, while red lines represent this average power of the CI-users (blue and red areas represent the SEM). Grey lines denote significant time-point differences between the two groups (Bonferroni corrected independent samples t-test, *p* < 0.05, only significance above 150 ms was considered).

We further contrasted the target and novel stimuli to test whether attention as signified by oscillatory activities were more directed toward target or toward novel stimuli. In the active condition, there was significant alpha- and beta reduction in this comparison (Fig. 5A), hence indicating stronger attentional process directed to target compared to novel stimuli. However, while in NH-listeners the significant alpha reduction was found in all ROIs (Fig. 5A a-d), in CI-users it was limited only to central ROI (Fig. 5A f). Indeed, as shown by the time course of alpha activity, the degree of this alpha reduction was significantly weaker in CI- compared to NH-listeners (Fig. 5B a-d). In the passive condition, there was no statistically significant differences in oscillatory activities between the two stimulus-types (not shown).

### Source localization: different source activations between NH-listeners and CI-users

**Fig. 6:**
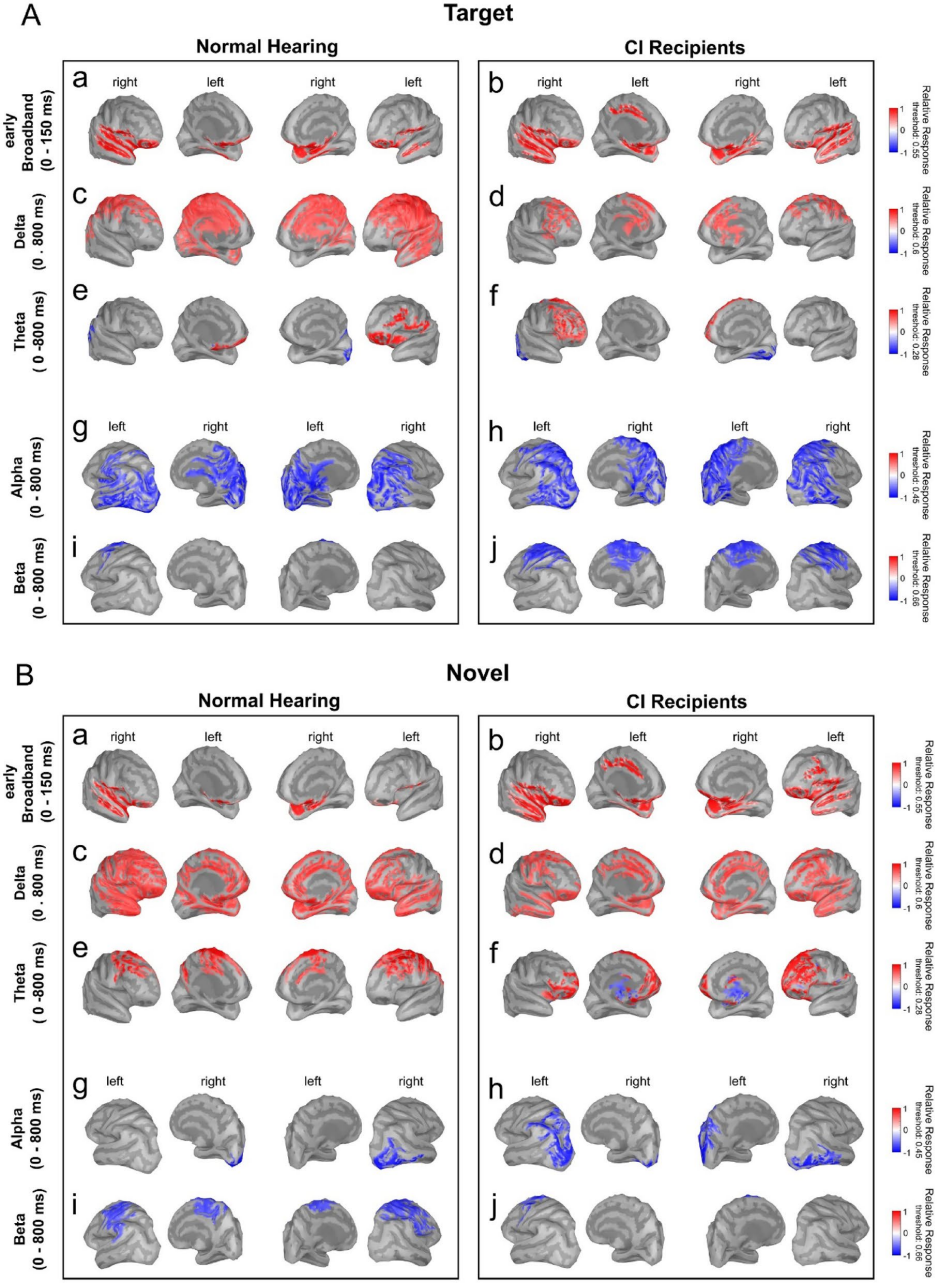
Brain sources of early N1 ERP component and oscillatory activities. Sources of the early broadband ERP component (at N1 latency) and oscillatory activities (delta, theta, alpha and beta) are given during target (**A**) and novel (**B**) processing in the active condition for NH-listeners and CI-users.

To understand the specific brain regions responsible for auditory attention, the sources of the neural activities during target (Fig. 6A) and novel processing (Fig. 6B) processing were reconstructed for the active condition. We focused on broadband N1 component along with the delta, theta, alpha and beta activities. The detailed brain sources for each neural activities for target and novel processing for both groups are listed in the subsequent table (Table 1).

**Table 1:** List of the brain sources of early N1 ERP component and oscillatory activities.

Target		
	NH-listeners	CI-users
N1 (0–150 ms)	Superior temporal gyrus Middle temporal gyrus	Superior temporal gyrus Middle temporal gyrus Left anterior cingulate gyrus
Delta (2-4 Hz; 0–800 ms)	Broad activity in temporal and parietal lobes	Right middle frontal gyrus Right inferior frontal gyrus (lateral area) Right superior frontal gyrus (medial area) Left superior parietal lobule Left inferior parietal lobule
Theta (5–7 Hz; 0–800 ms)	Left inferior frontal gyrus, Left inferior parietal lobule Left postcentral gyrus Left orbital gyrus	Right inferior frontal gyrus Right middle frontal gyrus
Alpha (8–12 Hz; 0–800 ms)	Inferior parietal lobule Medioventral occipital cortex Middle temporal gyrus Precuneus Posterior cingulate cortex	Inferior parietal lobule Medioventral occipital cortex Middle temporal gyrus Precuneus Posterior cingulate cortex Postcentral gyrus Superior frontal gyrus (medial area)
Beta (13–29 Hz; 0–800 ms)	Postcentral gyrus	Inferior parietal lobule, Postcentral gyrus Superior frontal gyrus (medial area)

Novel		
N1 (0–150 ms)	Superior temporal gyrus Middle temporal gyrus	Superior temporal gyrus Middle temporal gyrus Medial prefrontal cortex Left middle frontal gyrus Left anterior cingulate gyrus
Delta (2-4 Hz; 0–800 ms)	Broad activity in frontal and temporal lobes Precuneus Cingulate gyrus	Broad activity in frontal and temporal lobes Precuneus Cingulate gyrus
Theta (5–7 Hz; 0–800 ms)	Precentral gyrus Postcentral gyrus Inferior parietal lobule	Inferior frontal gyrus Middle frontal gyrus Orbital gyrus
Alpha (8–12 Hz; 0–800 ms)	Inferior temporal gyrus Medioventral occipital cortex	Inferior temporal gyrus Medioventral occipital cortex Inferior parietal lobule
Beta (13–29 Hz; 0–800 ms)	Inferior parietal lobule Postcentral gyrus Superior frontal gyrus (medial area)	Postcentral gyrus

Source-level neural activities: larger and sustained late activity in left temporal cortex for NH-listeners.

In summary, during **target** processing the sources of the neural activities differed between CI-users and NH-listeners as follows:

i. Beside the expected temporal cortex, CI-users exhibited additionally left cingulate gyrus as a source of N1.
ii. CI-users showed more local delta sources, especially in frontal gyrus and parietal lobule. NH-listeners had broader delta sources including large portions of temporal and parietal lobes.
iii. There was hemispheric difference in the sources of theta activity between the two groups: CI-users showed more right hemispheric activation of the frontal region, while NH-listeners had more left hemispheric activation of the temporal region.
iv. The source of beta during target processing was more pronounced in CI-users compared to NH-listeners, especially in the central region of the right hemisphere.

Furthermore, during **novel** processing the sources of the neural activities differed between CI-users and NH-listeners as follows:

i. Similar to N1-sources of target stimuli, CI-users exhibited in addition to temporal cortex, also the cingulate gyrus along with left middle frontal cortex as sources of N1.
ii. For the theta activity, CI-users showed more frontal region activation, while NH-listeners showed more activation in the parietal region.
iii. CI-users exhibited additional alpha source in the inferior parietal lobule, which not observed as alpha sources of the NH-listeners.
iv. The source of beta during novel processing was more pronounced in NH-listeners compared to CI-users, also in the central region of the right hemisphere.

### Source-level neural activities: larger and sustained late activity in left temporal cortex for NH-listeners

**Fig. 7:**
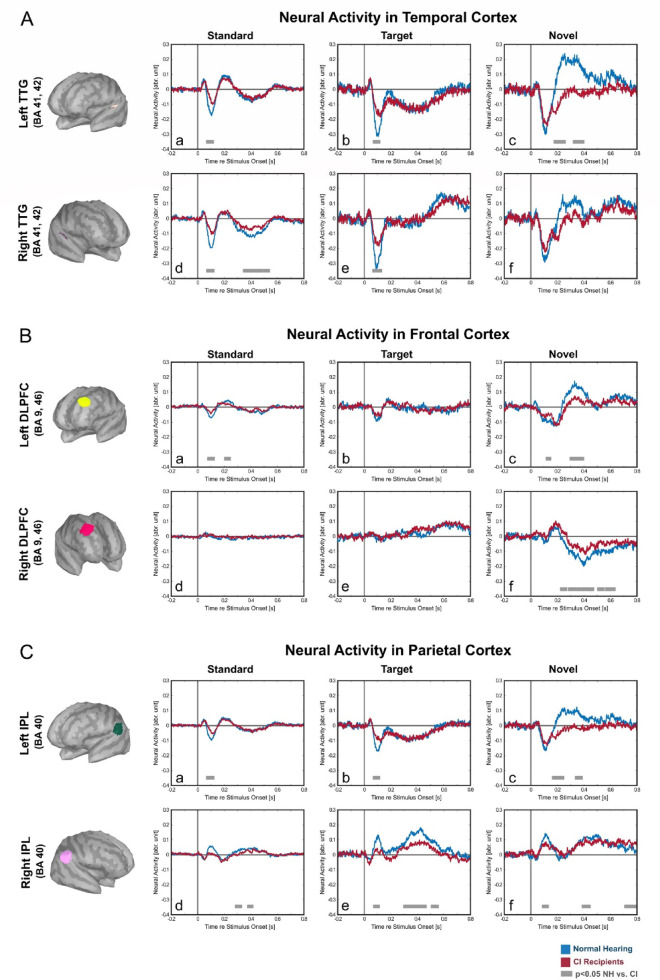
Neural activity in the bilateral temporal, frontal and parietal cortex. Estimated neural activity in TTG (transverse temporal gyrus) (**A**), DLPC (dorsolateral prefrontal cortex) (**B**) and IPL (inferior parietal lobules) (**C**) for Standard, Target and Novel during the active condition for NH-listeners (blue lines) and CI-users (red lines). Grey lines denote significant time-point differences between the two groups (Bonferroni corrected independent samples t-test, *p* < 0.05, only significance above 12 ms was considered).

For three distinct brain regions of the bilateral temporal-, frontal- and parietal lobe, the neural activity in the active condition was estimated for each stimulus-types (standard, target and novel) and each group (NH-listeners and CI-users) (Fig. 7). Specifically, these brain regions are (i) transverse temporal gyrus (TTG) including Brodmann area (BA) 41, 42, (ii) dorsolateral prefrontal cortex (DLPFC) or BA9, 46 dorsal area and (iii) inferior parietal lobule (IPL) or BA40 caudal area. These regions were chosen because of their involvement in early sensory auditory processing, central executive and attentional networks.

In the **temporal cortex**, the most prominent neural activities for all stimulus-types and both groups were observed in the first 100 ms post-stimulus as a complex of positive–negative peaks (Fig. 7A a-f). For both the standard and target, as expected, the early first negative peaks were larger in NH-listeners compared to CI-users (Fig. 7A a, b, d, e). Interestingly, for the novel stimuli, there was no significant difference in this early peak, but the NH-listeners showed a pronounced late positive peaks (198—246 ms) in the left temporal cortex, which was absent in CI-users (Fig. 7A c).

In the **frontal cortex**, the main responses were elicited by the novel stimuli with a clear lateralization effect between the two hemispheres (Fig. 7B c, f). For the novel stimuli, the main groups’ differences were found in the right frontal cortex (Fig. 7B f), which was the larger late sustained negative activity (300—464 ms) in NH-listeners compared to that in CI-users. Another significant difference between the two groups was also observable in the left frontal cortex for the novel stimuli (Fig. 7B c), where NH-listeners showed larger a larger late positive peak (310—392 ms) compared to CI-users. For target and standard stimuli, groups’ differences were minor and a statistical difference can only be found in left frontal cortex during standard processing (Fig. 7B a).

The **parietal cortex** exhibited also a lateralization effect, where for all stimulus-types the activities in the left cortex (Fig. 7C a, b, c) had opposite polarity than those in the right cortex (Fig. 7C d, e, f). A significant group difference was found during target processing in the right parietal cortex (Fig. 7C e), where NH-listeners elicited a larger late (312—456 ms) positive peaks compared to CI-users. Another significant difference was found during novel processing in the left parietal cortex (Fig. 7C c), where the NH-listeners showed a late positive peak (398—436 ms), which was absent in CI-users. This pattern of activity of the left parietal cortex resembled the activity of the left temporal cortex (compare Fig. 7A c). There were other significant group differences in the parietal cortex, but they appeared rather in short durations (Fig. 7 C a, b, d, f).

## Discussion

The current well-powered study (74 participants) extended the previous ERP study^26^ and investigated oscillatory signatures of attentional processing in CI-users and in a NH-listeners. It utilized an established three-stimulus auditory oddball paradigm, in both an active- and a passive listening condition (Fig. 1A) to differentiate attentional processing between unattended and attended task condition. As expected, these oscillatory activities were prominent in the active but not in the passive condition. This signified their close link with the top-down aspect of processing^26,60,61^. Both CI-users and NH-listeners elicited these pronounced activities for target and novel stimuli (Fig. 1B). During target processing, alpha desynchronization was prominent (Fig. 1C b,h, 2A). This supports the expected functional role of alpha desynchronization as a reflection of the attentional focus to task-relevant stimuli^33,62^. In line with this, alpha sources emerged in the parietal region, which is closely associated with the attention networks^63^. The degree of alpha reduction was stronger in NH-listeners for target stimuli (Fig. 2B e–h), indicating a more effective recruitment of task-relevant attention in NH-listeners compared to CI-users. Compared to NH-listeners, CI-users may demonstrate less efficient task-relevant attentional processes which may contributes to higher listening effort and prolonged response times^26^. For novel stimuli, although the group difference in alpha in the between-condition comparison was not pronounced (Fig. 2D), between-stimuli comparisons suggest that CI-users allocate more attention to task-irrelevant novel stimuli (Fig. 5, 6B g-h). Another prominent oscillatory signature was the perceptually driven delta-theta amplification (Fig. 2A, C) ^54,55^, which was as expected stronger in NH-listeners compared to CI-users during target processing (Fig. 2B b-d). Interestingly, during novel processing CI-users elicited a slightly stronger frontal delta-theta amplification (Fig. 2D a). This may reflect an overflow of the perceptual enhancement to the novel stimuli in CI-users^55,64^.

In the presence of frequent standard stimuli, attending to the target stimuli can be challenging due to an exogenous factor, namely the physical similarity between the target and standard stimuli. In target vs. standard in the active condition, there was little difference in alpha reduction between NH-listeners and CI-users (Fig. 3B e–h), suggesting that both groups utilized a similar extent of attention to the target. However, delta-theta amplification was weaker in CI-users compared to NH-listeners (Fig. 3B b-d). If interpreted as a signature of perceptual representation of the stimulus, weaker delta-theta amplification in CI-users implies that the perceptual discrimination of target might be less optimal in CI-users compared to NH-listeners. In our study, the standard and target stimuli were 600 Hz- and 756 Hz pure tones, respectively. Both fall within relatively narrow frequency range. While this discrimination is nonetheless achievable with CIs^65,66^, it involves less distinctive cues than in NH-listeners^3^. Depending on the implant manufacturers and the specific CI programming, both stimuli likely activated the same or if not neighboring electrode contact/s of the CI. It should be noted that the CI-group in this study included users from three major manufacturers. Nevertheless, for CI-users, stimulus discriminability depends primarily on temporal cues^3^. The NH-listeners, on the other hand, have both the cochlear place and the temporal code available. The reduced ability of CI-users in differentiating target from standard is further confirmed in the comparison of target vs. standard in the passive condition, for which only NH-listeners –but not the CI-users– exhibited a significant difference in neural response between target and standard stimuli (Fig. 3C a*; e–h*). This emphasized the robust ability of the NH-listeners in differentiating the two stimuli even in the unattended condition, but less of the CI-users. However, in the comparison of novel vs. standard in the passive condition, both groups show significant differences in oscillatory responses (Fig. 4C). This means that the contrast of novel vs. standard was apparent in the passive condition even for CI-users.

The comparison of novel vs. standard in the active condition suggested a group-difference in attentional processes to task-irrelevant stimuli, as we observed the occurrence of alpha reduction exclusively in CI-users (Fig. 4A g–h). This alpha reduction in CI-users lasted for almost the whole trial duration. Assuming alpha reduction reflects top-down active focused-attention directed to task-relevant stimuli, it is unusual to have this oscillatory signature in novel stimuli, which in this particular task should have been ignored. For CI-users the novel stimuli were unique, more complex and therefore more salient compared to the standard and the target stimuli. Especially with the particularly high synchrony of activity in the auditory nerve caused by the electric CI stimulus, the novel stimuli might have acted more as a distractor compared to the NH-listeners. This may elicit involuntary attention to this task-irrelevant stimulus due to its physical saliency^67^ and undermine the focused attention to the task-relevant target stimuli^50,68–70^. This may result in higher listening effort experienced by many CI-users^9^. Consistent with this interpretation, the CI-users showed less alpha reduction in target vs. novel comparison (i.e. a reduced alpha-power difference between these stimulus-types), which points to less focused-attention to the target stimuli in CI-users when compared to NH-listeners (Fig. 5). Several studies with selective attention paradigms have shown the close relation between alpha activity and distractor suppression^42,43^ in tasks with simultaneous presentation of distractor and target. Our results further emphasize that differences in attentional processing between CI-users and NH-listeners might not only be present during high-load tasks (e.g. selective attention task, listening in noise, audio-visual paradigm)^48,71,72^ but already occur in a simpler task requiring focused attention, such as in this three-stimulus oddball task.

Sources of the neural activities revealed further relevant differences between the two groups. Both the CI-users and the NH-listeners had, at first glance, comparable sources in regards of alpha- and beta desynchronization, especially in frontal- and parietal areas. Specifically, for the alpha band, these sources encompassed the inferior parietal lobule, the medioventral occipital cortex, the precuneus and the posterior cingulate cortex; areas for which are parts of attentional networks. CI-users, however, exhibited additional activation of the left parietal cortex during novel processing, which was not prominent in NH-listeners (Fig. 6B h), again supporting our interpretation of the higher attentional load in CI-users in processing the novel stimuli. Sources of early ERP in CI-users also included left anterior cingulate gyrus (Fig. 6A, B b), which was absent in NH-listeners. This area has been highlighted in its role in various cognitive functions, such as decision making, error monitoring and action adaptation^73–75^. Given the lower quality of the auditory input due to CI processing, the additional activation of the anterior cingulate cortex in CI-users might be necessary to transiently enhance responses to relevant acoustic stimuli in the auditory cortex^76,77^. The CI-users also exhibited more local delta activity sources and more frontal theta sources compared to NH (Fig. 6A, B c–f). This is consistent with reduced brain network global efficiency^78^ and greater recruitment of executive functioning, particularly in frontal areas^79,80^.

The source-level neural activities showed reduced responses in CI-users compared to NH in all three analyzed regions, namely the bilateral transverse temporal gyrus (TTG, Fig. 7A)^81,82^, the dorsolateral prefrontal cortex (DLPFC, Fig. 7B)^83,84^ and the inferior parietal lobules (IPL, Fig. 7C)^85^. These source-level neural activities consisted of two distinct responses: the early (below 150 ms) and the late responses (after 200 ms). Previous ERP studies have related the early auditory responses (i.e. N1, P2, N2) to stimulus detection and/or discrimination^12^, whereas the later responses (i.e. P3) are considered a reflection of higher-order cognitive processes including top-down attention^20^. The group difference in early source-level neural response in the temporal cortex during target processing thus confirmed the limitations of the CI-users in early stimulus processing when compared to NH-listeners (Fig. 7A b, e*)*. However, the early responses to the more complex novel stimuli did not differ significantly between the two groups (Fig. 7A c), which supports our earlier hypothesis that CI-users and NH participants did not differ in their ability to detect and discriminate the novel stimuli. Interestingly, the late response in the left temporal cortex to the Novel sound was stronger in NH-listeners compared to CI-users (Fig. 7A c). This may imply, specifically for the NH-listeners, higher-order influences (e.g. distractor suppression and/or predictive-based encoding) might already manifest at the lower hierarchy of the neural cascade (i.e. auditory cortex)^86^. Animal studies have shown that the late responses in the auditory cortex are dominated by cortico-cortical (induced) activity, especially top-down influences from higher-order areas^87–90^. Regarding the left lateralization of this late response, the left auditory cortex has been found to be particularly adaptable in complex sounds processing^91,92^. Together with the finding of late activities in the bilateral DLPFC and left IPL (Fig. 7B c, f, C c), this result suggests that the late processing in auditory cortex further cascades into higher-order areas. These late responses in higher-order areas were weaker in CI-users and may signal the suboptimal suppression of task-irrelevant novel stimuli in CI-users.

During novel processing, we found that the early activity in the right DLPFC was modestly stronger in CI-users than in NH-listeners (Fig. 7B f). This indicates that CI-users may recruit central executive network (CEN) regions (e.g. right DLPFC) in order to compensate the reduced activation in the dorsal attention network (DAN) (e.g. left IPL) and to support the limited distraction-suppression process. On the other hand, this altered processing may result in increased listening effort^93–97^. While the frontal neural activity was robust for the novel stimuli in both groups (Fig. 7B c, f*)*, the neural activity for the target stimuli was only marginal and did not differ significantly between NH-listeners and CI-users (Fig. 7B b, e). The main group differences between NH-listeners and CI users were found significant in the right parietal region (i.e. IPL, Fig. 7C e), where the CI-users showed weaker activation in both early- and late-responses. This region is a part of the ventral attentional network (VAN)^63^ and plays a role to reorient attention to the task-relevant stimuli and to respond accordingly^98,99^. Our result hint on less utilization of this region in CI-users compared to NH-listeners. This interpretation is further supported by the polarity reversal between the responses in left and right cortical parietal and frontal regions related to CEN, DAN and VAN networks^100^.

To investigate the possible effect of stimulation side on the overall neural activity pattern, we resorted CI-users into sub-groups (i.e. those receiving left ear stimulation and those receiving right ear stimulation; Supp. Figure 1). The result demonstrated that the late activities observed in NH-listeners was not present in either sub-group. Together with the fact that both CI-users and their age-matched NH-listeners were stimulated on the same ear, it can be concluded that side of stimulation did not account for the observed difference between CI-users and NH-listeners (Fig. 7A, C). For the lateralization pattern, a possible contributor may be the solely right-hand response of this particular task. However, this is unlikely, given that the lateralization effect was also found for standard and novel stimuli, for which no button presses were performed. To further explore lateralization effect, future studies may employ bilateral stimulation in unilateral CI-users, particularly to investigate as well the influence of implant side on oscillatory dynamics.

Taken together, the data imply that in CI-users, the smeared representation of the sensory input complicates stimulus discrimination, but also has downstream effects on how attention is recruited to enhance the task-relevant stimulus and suppress the task-irrelevant stimulus.

## Conclusion

The present study examined the auditory cognitive processing in cochlear implant users (CI-users) by combining time–frequency analysis and source localization methods to find neural oscillatory correlates of attentional processing. The most prominent oscillatory activities were delta-theta synchronization, alpha- and beta desynchronization, mainly observed in active condition. The difference between CI-users and normal hearing listeners (NH-listeners) was pronounced in delta-theta activity and might reflect a group difference in perceptual processing of the stimuli. Interestingly, while attention-related alpha activity was robust for the task-relevant target stimulus, CI-users exhibited attentional alpha signature also during novel processing, suggesting attentional allocation to the task-irrelevant stimulus. Consistent with this, CI-users also exhibited broader sources of alpha activity during novel processing. The local neural activities in the transverse temporal gyrus (TTG), in the dorsolateral prefrontal cortex (DLPFC) and in the inferior parietal lobule (IPL) were, however, weaker in CI-users and were consistent with different recruitment of ventral and dorsal attentional networks. Overall, the result suggests that CI-users may direct their attention toward task-irrelevant stimuli and thereby impeding their focus on task-relevant stimuli, thus may increase their listening effort.

## Methods

The current study was performed in accordance with the Declaration of Helsinki and approved by the Ethics Committee of the Hannover Medical School (vote number 7425). All participants provided written informed consent prior to the experiments and all received a reimbursement for their participation in this study.

### Participants

The dataset of the present work was collected in conjunction with the previous study^26^, and has not been analyzed nor published previously. The participant sample was identical with the previous study^26^. It comprised of forty post-lingual cochlear implant users (CI-users) (27 female; age: 28–79 years, 59.4 years (Mean (*M*)) ± 1.5 years (Standard Error of the Mean (*SEM*)) and forty normal hearing listeners (NH-listeners) (27 female; age: 29–77 years, 60 years (*M*) ± 1.6 years (*SEM*)). Seventy-four participants were right-handed, one participant was left-handed and the remaining five participants were ambidextrous according to the Edinburgh Handedness Inventory^101^. NH-listeners and CI-users did not differ in terms of duration of education. Eighteen CI-users were implanted unilaterally (nine right-implanted and nine left implanted) and twenty-two bilaterally. In bilaterally implanted CI-users, the better performing CI side was investigated which was defined on the basis on the speech audiometry performed in the last clinical check. In sum, twenty-two CI-users were examined on the left implanted side, and eighteen CI users on the right implanted side. Nineteen CI-users used cochlear implants manufactured by Cochlear, twelve used implants from Advance Bionic and nine from MED-EL. All CI-users had been using their device continuously for at least 10 months. For details of CI-users (implant systems, demographic information and speech recognition tests) please refer to previous study^26^. NH-listeners had an average pure tone thresholds of less than or equal to 30 dB HL.

### Stimuli and procedure

As described in the original study^26^, for the experiment, participants sat comfortably in acoustically and electromagnetically shielded booth and performed an auditory three-stimulus oddball paradigm^102^. The oddball paradigm included three stimulus-types: standard, target and novel stimuli. The standard stimuli consisted of a 600 Hz pure tone, with an occurrence probability of 70%. The target stimuli comprised of a 756 Hz pure tone with an occurrence probability of 15%. The novel stimuli consisted of a set of trial-unique environmental sounds (e.g. a dog bark, a telephone ringing or a car engine noise) with an occurrence probability of 15%^103^. The experimental paradigm was identical to the one reported previously^26^, with the only difference being a longer stimulation duration (400 ms instead of 200 ms) to allow more sustained neural responses and thus improved signal-to-noise ratios of the signals^104^, especially the low-frequency components (e.g. delta-theta band), while preserving the high-frequency components (e.g. alpha-, beta bands). The oddball paradigm was performed in two conditions, in particular an active and a passive condition, where the former required participants to respond to the target via a button press on a typical computer keyboard. Each condition comprised 300 trials (210 standard, 45 target and 45 novel stimuli). The passive condition always followed the active condition to ensure an optimum of attentional state in the active condition. The inter-stimulus interval varied randomly according to a uniform distribution (800, 900 or 1000 ms). All stimuli were initially presented at an acoustic reference level (~ 65 dB SPL). To ensure comparable perceived loudness across both groups, sound level was then finely adjusted individually based on a seven-point loudness scale (“not heard”, “very soft”, “soft”, “moderate”, “loud”, “very loud” and “too loud”)^26,105,106^ to the “moderate” level, which is equivalent to a level of 60–70 dB(A) of the perceived loudness of the normal hearing listeners^107,108^. Therefore, although the final stimulation levels were not identical between the NH- and CI-groups in term of physical units, they were closely matched in terms of perceived loudness. For CI-users, the auditory stimulation was delivered via a direct audio cable connection to the CI speech processors (twenty-two left ear, eighteen right ear). NH-listeners received monaural auditory stimulation via an inserted earphone at the ear side corresponding to that of their matched CI participant (twenty-two left ear, eighteen right ear).

### Data acquisition

Continuous EEG data were recorded using 78 Ag/Ag–Cl scalp electrodes using a 128-channels Quik-Cap (Compumedics, Neuroscan) and a SynAmp amplifier system (Compumedics, Neuroscan)^26^. Electrodes were placed according to the 10–10 system, with seven additional electrodes over posterior cortical regions. Two additional electrodes were placed at left and right mastoids. Horizontal and vertical eye movements were recorded by additional electrodes placed at the outer canthi of both eyes and below and above one eye, respectively. A reference electrode was placed at the nose-tip. A midline electrode, which was located slightly anterior to Fz, served as ground electrode. Data were recorded at a sampling rate of 1 kHz and without online filtering using the software Curry Neuroimaging Suite 7 (Neuroscans, Compumedics). Impedances were maintained below 20 kΩ prior to data acquisition.

### Data analysis

The EEG data were offline analyzed using the Brainstorm toolbox^109^, the FieldTrip toolbox^110^ and custom-made Matlab scripts (Mathworks Inc.). EEG data from six participants were excluded due to a defect in the raw dataset (NH: two participants, CI: two participants) or due to poor hit-rates in the active condition (NH: one participant, CI: one participant).

### Preprocessing

The raw EEG data were imported in Brainstorm for preprocessing procedures. The preprocessing was band-pass- and notch filtering, segmentation and artifact removal, all performed in Brainstorm. The EEG data were first filtered with a 0.02–250 Hz even-order phase FIR (Finite Impulse Response) band-pass filter based on a Kaiser window design, which compensate for the filter delay by shifting the sequence backward in time to achieve zero-phase and zero-delay. Following that, a second order IIR (Infinite Impulse Response) 50 Hz notch filter with zero-phase lag was applied to the data^111^ to remove the electrical line noise. Independent component analysis (ICA) weight matrix was calculated with the filtered data. Independent components reflecting artifacts such as eye movements, eye blinks, electrical heartbeat activity, as well as other sources of non-cerebral activity were identified and removed from the data. Additionally, independent components reflecting electrical CI artifacts were identified and removed (for representative samples, see supplementary materials). On average, there were additional six components related to CI artifacts removed for the CI-users. Additionally, automatic artifact rejection modules based on SSP (Signal-Space Projection) in Brainstorm were applied to the data to detect blink and eye-movement artifacts^112,113^. The resulting time periods of the artifacts were marked to be identified and the potentially contaminated epochs were removed. The data were then segmented into 2000 ms epochs and triggered by the stimulus onset (0: stimulus onset, -1000 ms pre-stimulus and 1000 ms post-stimulus). Epoch by epoch inspection was then performed to further identify and remove epochs with non-stereotyped artifacts. In average, 68% of trials were kept for further analysis.

### Event-related potential

The preprocessed trials for each stimulus-type (standard, target and novel stimuli) and each condition (active and passive condition) were filtered with an additional 1–40 Hz even-order phase FIR (Finite Impulse Response) band-pass filter, comparable to that implemented in the previous study^26^. Single-subject event-related potentials (ERP) were calculated for each stimulus-type and for each condition. Grand-mean ERPs were then calculated for each group (NH and CI) by averaging the single-subject ERPs of the respected subjects in each group. The resulting grand-mean ERPs were then baseline corrected (50–150 ms pre-stimulus) and they were pooled in a region-of-interest (ROI) including central electrode positions (i.e. Central ROI: C1, Cz, C2, CP1, CPPz, CP2). This was done for each group, for each stimulus-type and for each condition. The end result of the analysis was a grand-mean ERP (central ROI) for each group and for each stimulus-type in each condition. For details of the ERP analysis and the results, please refer to previous study^26^.

### Time–frequency analysis

Time–frequency representations (TFRs) of the preprocessed trials were calculated for each stimulus-type (standard, target and novel stimuli) and each condition (active and passive condition). This was done using a Wavelet transformation in Brainstorm. The predefined parameters of the Morlet wavelet in Brainstorm were chosen for this analysis (central frequency: 1 and time resolution: 3). Frequencies from 1 to 90 Hz were analyzed by means of 1 Hz linear steps. The resulting TFR was normalized to the baseline period (-350 to -50 ms) and extracted for the time period of interest (-400 to 800 ms post-stimulus). The observed oscillatory activity was defined as *synchronization*, when its power increased relative to baseline, and *desynchronization* when its power decreased relative to baseline. The complex TFRs of the trials were then imported to Matlab workspace and processed further in Matlab. This step allowed us to logarithmically plot the TFRs and to perform cluster-based statistical tests using the Fieldtrip toolbox^114^. Afterwards, the complex TFRs of the single trials (for each stimulus-type in each condition) were pooled in four different ROIs before they were averaged throughout the trials: 1) Frontal ROI: F1, Fz, F2, FC1, FCz, FC2; 2) Central ROI: C1, Cz, C2, CP1, CPPz, CP2; 3) Parietal ROI: PPO1, PPO2, PO3, PO1, POz, PO2, PO4, POO3, POO4 and 4) Occipital ROI: OI1, OI2, I1, Iz, I2, POO11h, POO12h. The result of all of the previous steps was one TFR for each ROI and for each stimulus-type in each condition. In a next step, the TFRs were averaged together according to the corresponding group (NH-listeners or CI-users). In order to compare the stimulus-types and conditions, difference-TFRs were calculated by subtracting TFRs from one condition/stimulus-type from another condition/stimulus-type (e.g. *active vs. passive* means: TFRs of active minus TFRs of passive condition; similarly *Target vs. Standard* means: TFRs of Target minus TFRs of Standard). Cluster-based permutation test^115^ was performed to define statistically significant oscillatory activity differences between the two TFRs which were also plotted as black contours on the respective difference-TFRs (see 2.4.7 Statistical tests for details). If no significant cluster was found, the difference-TFR would be marked ‘ns’. The difference of oscillatory activity between two stimulus-types/conditions was labeled as *amplification*, when its power increased relative to the other stimulus-type or condition (e.g. active vs. passive, Target vs. Standard), and *reduction* when its power decreased relative to the other type/condition.

### Time-course of oscillatory activities

To visualize the power fluctuations of oscillatory activity in the specific frequency bands and compare them between NH-listeners and CI-users, the power from the difference-TFRs for each time step was averaged across the frequency of interest (delta-theta: 2–7 Hz, alpha: 8–12 Hz and beta: 13–29 Hz) and was plotted for the whole trial duration (-400 to 800 ms). Delta- and theta activity were pooled together as “delta-theta activity” since in most of the TFRs only a single oscillatory activity was observed in these frequency bands (2–7 Hz). The time-course of the activity was statistically compared between the two groups using Bonferroni corrected t-tests (alpha < 0.05, corrected alpha = 0.000125; see 2.4.7 Statistical tests for details). The latencies, for which the most significant (longest duration) group differences were observed, were listed along with the peak-power changes of each group in those latencies in the corresponding tables in the supplementary materials.

### Source localization

Because most of the oscillatory activities were observed during target and novel processing in active condition, we estimated using Brainstorm the sources of these activities^109,116,117^.For each subject, channel by channel was inspected and removed when it contained substantial amounts of artifact in the continuous recording. For the CI-users, these were mainly channels surrounding the implanted ears which were contaminated with CI artifacts. Using the remaining clean channels, a head model (containing dipoles) was computed for each subject using a symmetric Boundary Element Method from the OPENMEEG software^118^. The preprocessed trials during the pre-stimulus period for each stimulus-type were then used as input to calculate the noise covariance matrix. In addition to the noise covariance matrix, we also calculated the data covariance matrix during the activation period, which is required for applying linear-constraint minimum variance (LCMV) beamforming method^119^. Using the LCMV beamforming method, the source space time-series neural activity (Pseudo Neural Activity Index (PNAI)) could then be estimated for each dipole in the source space. The resulting source space activity was then averaged across all subjects for each group (NH-listeners or CI-users). The grand-mean source space activity was then averaged for the time period 0—150 ms post-stimulus to obtain the estimated source of the early broadband ERP signal (i.e. N1 component).

To estimate the sources of oscillatory activities, we performed time–frequency decomposition of the calculated source space activity. Due to computational limitation, we used the Hilbert transformation instead of the wavelet transform to calculate the power for frequency-band averages of the source space activity (delta: 2–4 Hz, theta: 5–7 Hz, alpha: 8–12 Hz and beta: 13–29 Hz) for the whole duration of activation period (0—800 ms). The resulted oscillatory activity in the source space was then averaged across the subjects for each group (NH-listeners or CI-users). For the visualization of the grand-mean source space activities, minimal cluster sizes and thresholds were kept the same across stimulus-types and groups to ensure reliable comparison.

### Source-level neural activities

We used Brainstorm to estimate the neural activities from brain sources related to standard, target and novel processing in the active condition^109,116,117^. Specifically, we utilized Brainnetome atlas^120^, to define specific brain regions of interest (also called scouts in Brainstorm). We focused this analysis on three distinct regions related to auditory attention, namely (i) the bilateral transverse temporal gyrus (TTG; primary auditory cortex; Heschl’s gyrus; Brodmann area (BA) 41 and 42), (ii) the dorsolateral prefrontal cortex (DLPFC; middle frontal gyrus; BA 9 and BA 46) and (iii) the inferior parietal lobules (IPL; BA 40)^81–84,121–123^. These regions were chosen because each represents a major locus of the early sensory auditory processing, central executive and attentional networks. For the bilateral transverse temporal gyrus, the provided scouts from the Brainnetome atlas (consisted of 54 vertices and covered 8.25 cm^2^) were used as they are. For the dorsolateral prefrontal cortex and inferior parietal lobule, the size of each scout was increased to 152 vertices covering around 20 cm^2^ (left frontal: 19.29 cm^2^, right frontal: 20.58 cm^2^, left parietal: 23.32 cm^2^, right parietal: 24.15 cm^2^) to also include the rostral area of the prefrontal cortex and the caudal area of the inferior parietal lobule. For each scout of each stimulus-type and each group, the estimated source space time-domain activity from the previous analysis (2.4.5. Source localization) was averaged using a Brainstorm function. The averaged neural activity for each scout was statistically compared between the two groups using Bonferroni corrected t-tests (alpha < 0.05, corrected alpha = 0.00025; see 2.4.7 Statistical tests for details). The latencies, for which the most significant (longest duration) group differences were observed, were listed along with the peak-amplitudes of the activities of each group in those latencies in the corresponding tables in the supplementary materials. Additionally, to assess whether the side of ear affected lateralization pattern, source-level neural activity during novel processing in left TTG and left IPL was computed separately for CI-users with only left- and those with only right-ear stimulation (see supplementary materials).

### Statistical tests

The time–frequency data were statistically analyzed using a non-parametric cluster-based permutation approach using the Fieldtrip toolbox^115^. Independent samples t-tests between two stimulus-types/conditions were calculated for each sample point. Significant values (alpha < 0.05) were clustered based on their adjacency in time and frequency. The critical p-value for each cluster was calculated using the Monte Carlo method with 500 random permutations. If the summed t-value of the observed data cluster was higher than 95% of the random partitions, then the cluster was considered to represent a significant difference between the two compared TFRs. The cluster was plotted as black contours on the respective difference-TFRs. If no significant cluster was found, the difference-TFR would be marked ‘ns’. If significant clusters in the frequency band of interest (delta-theta: 2–7 Hz, alpha: 8–12 Hz and beta: 13–29 Hz) was found in at least one group (NH or CI), the time-course of the oscillatory activity (power average in that frequency band of interest) was calculated and plotted for both groups. For each 2 ms time point, the oscillatory activity was statistically compared between the two groups using independent samples t-tests (alpha < 0.05). As the tests may result in false positives due to multiple comparisons, Bonferroni correction was applied (corrected alpha = 0.000125). Furthermore, since EEG signals are highly temporally correlated^124,125^, significance was only considered if it exceeded a minimal time window threshold of 150 ms. The source-level neural activities were also statistically compared between the two groups using independent samples t-tests (alpha < 0.05). The source-level neural activity for each group and each scout was statistically compared using independent samples t-tests firstly to the baseline (zero activity), before performing the group comparison also using independent samples t-tests. Because this analysis yield more sensitive results, we used 4 ms time bin (instead of 2 ms), which resulted in a corrected alpha of 0.00025. In this comparison, significance was considered if it exceeded a minimal time window threshold of 12 ms.

## Supplementary Information

Below is the link to the electronic supplementary material.

Supplementary Material 1

## Data Availability

The de-identified EEG data can be made available upon reasonable request to the corresponding author, subject to institutional and ethical approvals.

## Code availability

Customized Matlab scripts for EEG processing in this study are available on GitHub at: https://github.com/brllant
