# Supplementary material for: Alpha oscillatory activity reveals focused-attentional disparity between cochlear implant users and normal hearing listeners
Source: Sci Rep. 2026 May 9;16:14690. doi: 10.1038/s41598-026-52434-6 (PMC13157490; doi:10.1038/s41598-026-52434-6)
Supplement: Supplementary file 1 — Supplementary Material 1 [file 41598_2026_52434_MOESM1_ESM.docx]

**Supplementary materials**

**Active vs. passive:**

| **Target**  **active minus passive** | | | | | | | |
| --- | --- | --- | --- | --- | --- | --- | --- |
|  | **Delta-Theta amplification**  **(2-7 Hz)** | | | **Alpha reduction**  **(8-12 Hz)** | | | **Beta reduction**  **(13-29 Hz)** |
|  | **Signifi-cant latency** | **NH power** | **CI power** | **Signifi-cant latency** | **NH power** | **CI power** | ns |
| **Frontal** | ns | ns | ns | **394 ms**  **–**  **630 ms** | **-1.261 dB** | **-.781 dB** |  |
| **Central** | **188 ms**  **–**  **428 ms** | **.961**  **dB** | **.751**  **dB** | **462 ms**  **–**  **604 ms** | **-2.015**  **dB** | **-1.601**  **dB** |  |
| **Parietal** | **0 ms**  **–**  **624 ms** | **.754**  **dB** | **.440**  **dB** | **414 ms**  **–**  **630 ms** | **-1.934 dB** | **-1.466 dB** |  |
| **Occipital** | **0 ms**  **–**  **544 ms** | **.543**  **dB** | **.425**  **dB** | **0 ms**  **–**  **604 ms** | **-1.456 dB** | **-1.052 dB** |  |
| **Novel**  **active minus passive** | | | | | | | |
|  | **Delta-Theta amplification**  **(2-7 Hz)** | | | **Alpha reduction**  **(8-12 Hz)** | | | **Beta reduction**  **(13-29 Hz)** |
|  | **Signifi-cant latency** | **NH power** | **CI power** | ns | | | ns |
| **Frontal** | **222 ms**  **–**  **404 ms** | **.736**  **dB** | **.852**  **dB** |  |  |  |  |
| **Central** | **624 ms**  **–**  **800 ms** | **.323**  **dB** | **.167**  **dB** |  |  |  |  |
| **Parietal** | **158 ms**  **–**  **732 ms** | **.473**  **dB** | **.365**  **dB** |  |  |  |  |

***Supplementary Tab. 1: For the active vs. passive comparison, the main group differences between NH-listeners and CI-users were in the delta-theta and alpha activity during target processing, and in the delta-theta during novel processing.*** *This table lists the latencies at which the most significant group differences in the oscillatory effects were found; and the peak-power changes at those latencies (maximum for amplification and minimum for reduction; ns means no significance difference found between the two groups). This table corresponds to Fig. 2 of the main text.*

**Target vs. standard:**

| **Target minus Standard**  **Active** | | | | | | | |
| --- | --- | --- | --- | --- | --- | --- | --- |
|  | **Delta-Theta amplification**  **(2-7 Hz)** | | | **Alpha reduction**  **(8-12 Hz)** | | | **Beta reduction**  **(13-29 Hz)** |
|  | **Signifi-cant Latency** | **NH Power** | **CI Power** | **Signifi-cant Latency** | **NH Power** | **CI Power** | ns |
| **Frontal** | ns | ns | ns | **394 ms –**  **582 ms** | **-1.052 dB** | **-.701 dB** |  |
| **Central** | **226 ms –**  **428 ms** | **1.092**  **dB** | **.956**  **dB** | ns | ns | ns |  |
| **Parietal** | **156 ms –**  **486 ms** | **.862**  **dB** | **-732**  **dB** | ns | ns | ns |  |
| **Occipital** | **468 ms –**  **800 ms** | **.413**  **dB** | **.859**  **dB** | ns | ns | ns |  |
| **Target minus Standard**  **Passive** | | | | | | | |
|  | **Delta-Theta amplification**  **(2-7 Hz)** | | | **Alpha amplification**  **(8-12 Hz)** | | | **Beta reduction**  **(13-29 Hz)** |
|  | ns | | | **Signifi-cant Latency** | **NH Power** | **CI Power** | ns |
| **Frontal** |  |  |  | **.032 ms –**  **800 ms** | **.352**  **dB** | **.061**  **dB** |  |

***Supplementary Tab. 2: For target vs. standard comparison, the main group differences between NH-listeners and CI-users regarding their oscillatory activities were found in the delta-theta and the frontal alpha activity in both, the active and passive condition. Notice that the direction of alpha effects was reversed in the active (reduction) compared to the passive (amplification) condition.*** *This table lists the latencies at which the most significant group differences in the oscillatory effects were found; and the peak-power changes at those latencies (maximum for amplification and minimum for reduction; ns means no significance difference found between the two groups). This table corresponds to Fig. 3 of the main text.*

**Novel vs. standard:**

| **Novel minus Standard**  **active** | | | | | | | |
| --- | --- | --- | --- | --- | --- | --- | --- |
|  | **Delta-Theta amplification**  **(2-7 Hz)** | | | **Alpha reduction**  **(8-12 Hz)** | | | **Beta reduction**  **(13-29 Hz)** |
|  | **Signifi-cant latency** | **NH power** | **CI power** | **Signifi-cant latency** | **NH power** | **CI power** | ns |
| **Frontal** | **226 ms –**  **390 ms** | **1.310**  **dB** | **1.182**  **dB** | **392 ms –**  **656 ms** | **-.212 dB** | **0**  **dB** |  |
| **Central** | **216 ms –**  **422 ms** | **1.169**  **dB** | **1.032 dB** | ns | ns | ns |  |
| **Parietal** | **214 ms –**  **560 ms** | **.866**  **dB** | **.752**  **dB** | **312 ms –**  **800 ms** | **-.677**  **dB** | **-.978**  **dB** |  |
| **Occipital** | **252 ms –**  **416 ms** | **.759**  **dB** | **.711**  **dB** | **358 ms –**  **800 ms** | **-.452 dB** | **-.782 dB** |  |
| **Novel minus Standard**  **passive** | | | | | | | |
|  | **Delta-Theta amplification**  **(2-7 Hz)** | | | **Alpha amplification**  **(8-12 Hz)** | | | **Beta reduction**  **(13-29 Hz)** |
|  | **Signifi-cant latency** | **NH power** | **CIpower** | **Signifi-cant latency** | **NH power** | **CI power** | ns |
| **Frontal** | **0 ms**  **–**  **550 ms** | **.643**  **dB** | **.313**  **dB** | **98 ms**  **–**  **800 ms** | **.568**  **dB** | **.170**  **dB** |  |
| **Central** | **50 ms**  **–**  **538 ms** | **.581**  **dB** | **.357**  **dB** | **124 ms –**  **800 ms** | **.463**  **dB** | **.157**  **dB** |  |
| **Parietal** | **128 ms –**  **606 ms** | **.423**  **dB** | **.308**  **dB** | ns | ns | ns |  |
| **Occipital** | **50 ms**  **–**  **800 ms** | **.383**  **dB** | **.262**  **dB** | ns | ns | ns |  |

***Supplementary Tab. 3: For the novel vs. standard comparison, the main group differences between NH-listeners and CI-users were in delta-theta and alpha activity in both the active and passive conditions. While previously in active condition, group difference in the comparison target vs. standard in alpha were only modest (only frontal), here in the comparison novel vs. standard, group differences occurred in more ROIs (frontal, parietal and occipital).*** *This table lists the latencies at which the most significant group differences in the oscillatory effects were found; and the peak-power changes at those latencies (maximum for amplification and minimum for reduction; ns means no significance difference found between the two groups). This table corresponds to Fig. 4 of the main text.*

**Target vs. novel**

| **Target minus Novel**  **active** | | | | | |
| --- | --- | --- | --- | --- | --- |
|  | **Delta-Theta amplification**  **(2-7 Hz)** | **Alpha reduction**  **(8-12 Hz)** | | | **Beta reduction**  **(13-29 Hz)** |
|  | ns | **Signifi-cant latency** | **NH power** | **CI power** | ns |
| **Frontal** |  | **122 ms –**  **372 ms** | **-.704 dB** | **-.434 dB** |  |
| **Central** |  | **544 ms –**  **664 ms** | **-1.572**  **dB** | **-1.344 dB** |  |
| **Parietal** |  | **0 ms**  **–**  **800 ms** | **-1.611 dB** | **-1.034 dB** |  |
| **Occipital** |  | **0 ms**  **–**  **800 ms** | **-1.119**  **dB** | **-.557 dB** |  |

***Supplementary Tab. 4: For the target vs. novel comparison, the main group differences between NH-listeners and CI-users were found exclusively in the alpha activity and only in the active condition. Here, CI-users elicited weaker alpha reduction in target vs. novel compared to NH-listeners.*** *This table lists the latencies at which the most significant group differences in the oscillatory effects were found; and the peak-power changes at those latencies (maximum for amplification and minimum for reduction; ns means no significance difference found between the two groups). This table corresponds to Fig. 5 of the main text.*

**Source-level neural activities**

| **Early neural activity** | | | | | | | | | |
| --- | --- | --- | --- | --- | --- | --- | --- | --- | --- |
|  | **Standard** | | | **Target** | | | **Novel** | | |
|  | **Signifi-cant Latency** | **NH Ampli**  **-tude** | **CI Ampli**  **-tude** | **Signifi-cant Latency** | **NH Ampli**  **-tude** | **CI Ampli**  **-tude** | **Signifi-cant Latency** | **NH Ampli**  **-tude** | **CI Ampli**  **-tude** |
| **Left**  **TTG** | **76 ms**  **–**  **108 ms** | **-.171** | **-.099** | **78 ms**  **–**  **108 ms** | **-.312** | **-.185** | ns | ns | ns |
| **Right**  **TTG** | **76 ms**  **–**  **112 ms** | **-.198** | **-.107** | **76 ms**  **–**  **92 ms** | **-.350** | **-.185** | ns | ns | ns |
| **Left DLPFC** | **84 ms**  **–**  **116 ms** | **-.076** | **-.049** | ns | ns | ns | **126 ms –**  **154 ms** | **-.104** | **-.077** |
| **Right DLPFC** | ns | ns | ns | ns | ns | ns | **242 ms –**  **260 ms** | **.0710** | **.105** |
| **Left**  **IPL** | **88 ms**  **–**  **112 ms** | **-.101** | **-.057** | **78 ms**  **–**  **106 ms** | **-.173** | **-.115** | ns | ns | ns |
| **Right**  **IPL** | ns | ns | ns | **92 ms**  **–**  **108 ms** | **.126** | **.032** | **98 ms**  **–**  **112 ms** | **.138** | **.071** |

***Supplementary Tab. 5: Main group differences between NH-listeners and CI-users in their neural activities in the early time period were mainly in temporal cortex during standard and target, in frontal cortex during novel and in parietal cortex during target processing.*** *This table lists the latencies, when the most significant group differences in the source-level the neural activity of the bilateral TTG (transverse temporal gyrus), DLPC (dorsolateral prefrontal cortex) and IPL (inferior parietal lobules) between normal hearing and CI-users in the early time period were found; along with the peak-amplitudes of each group in those latencies (ns means no significance difference found between the two groups). This table corresponds to Fig. 7 (early period) of the main text.*

| **Late neural activity** | | | | | | | | | |
| --- | --- | --- | --- | --- | --- | --- | --- | --- | --- |
|  | **Standard** | | | **Target** | | | **Novel** | | |
|  | **Signifi-cant Latency** | **NH Ampli**  **-tude** | **CI Ampli**  **-tude** | **Signifi-cant Latency** | **NH Ampli**  **-tude** | **CI Ampli**  **-tude** | **Signifi-cant Latency** | **NH Ampli**  **-tude** | **CI Ampli**  **-tude** |
| **Left**  **TTG** | ns | ns | ns | ns | ns | ns | **198 ms –**  **246 ms** | **.236** | **.025** |
| **Right TTG** | **354 ms –**  **532 ms** | **-.128** | **-.082** | ns | ns | ns | ns | ns | ns |
| **Left DLPFC** | **212 ms –**  **236 ms** | **.042** | **.020** | ns | ns | ns | **310 ms –**  **392 ms** | **.169** | **.070** |
| **Right DLPFC** | ns | ns | ns | ns | ns | ns | **300 ms –**  **464 ms** | **-.189** | **-.116** |
| **Left**  **IPL** | ns | ns | ns | ns | ns | ns | **178 ms –**  **246 ms** | **.116** | **-.013** |
| **Right**  **IPL** | **296 ms –**  **320 ms** | **.025** | **.009** | **312 ms –**  **456 ms** | **.176** | **.094** | **398 ms –**  **438 ms** | **.117** | **.113** |

***Supplementary Tab. 6: Main group differences between NH-listeners and CI-users in their neural activities in the late time period were mainly in frontal and parietal cortex during novel processing.*** *his table lists the latencies, when the most significant group differences in the source-level the neural activity of the bilateral TTG (transverse temporal gyrus), DLPC (dorsolateral prefrontal cortex) and IPL (inferior parietal lobules) between normal hearing and CI-users in the early time period were found; along with the peak-amplitudes of each group in those latencies (ns means no significance difference found between the two groups). This table corresponds to Fig. 7 (late period) of the main text.*

**Neural activity in CI-Users with left- or right-ear stimulation**

*
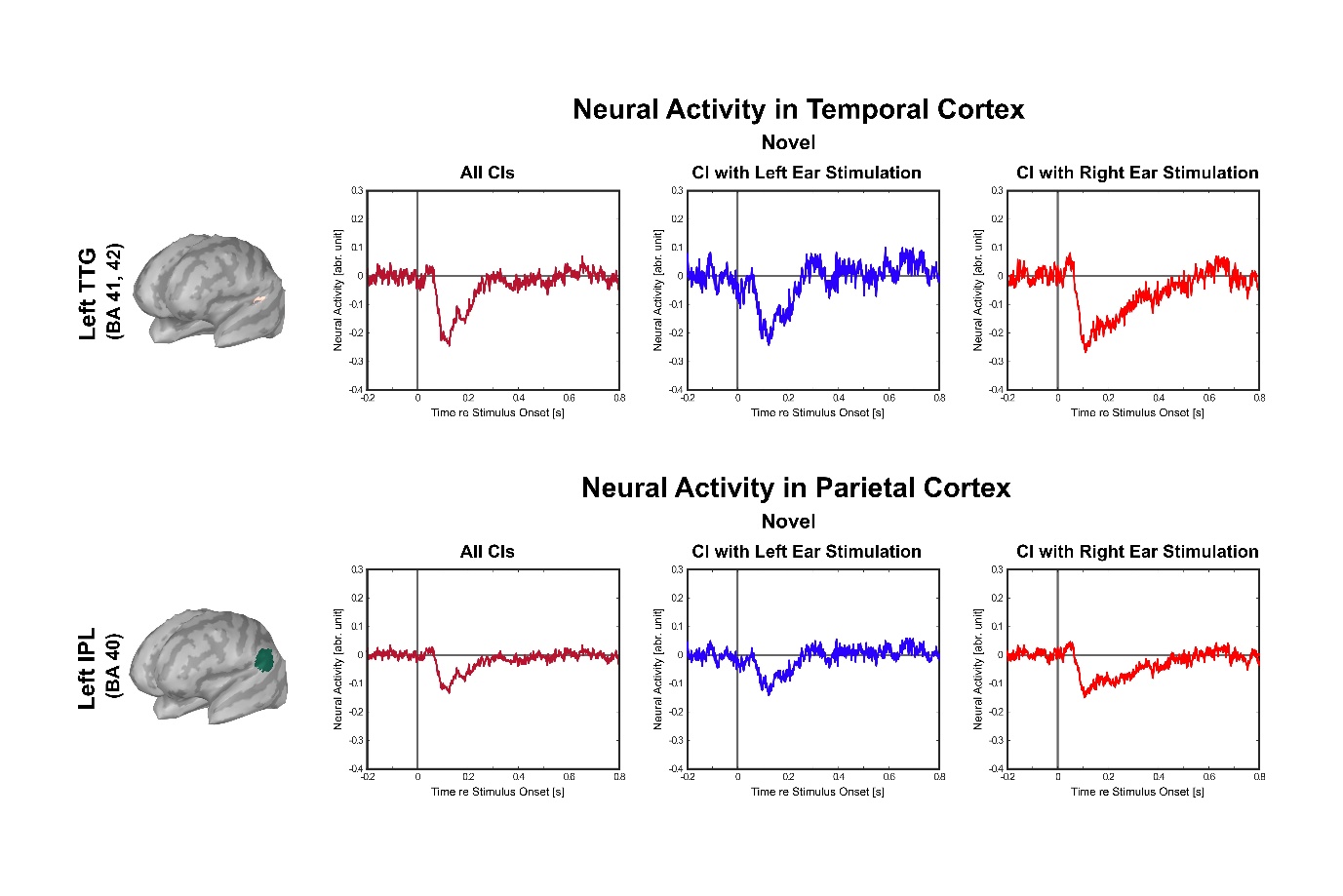
*

***Supplementary Fig. 1: Sub-group analysis of neural activity in left temporal and parietal cortex in CI-users.*** *Pronounce late activity during novel processing in left TTG and left IPL was observable in NH-controls, but not in CI-users (Fig. 7 of the main manuscript). To test whether this was due to different ears being tested across participants, the neural activity during novel processing in these two regions was plotted separately for CI-users with only left- and those with only right-ear stimulation. Regardless of which ear was stimulated, no late activity was observable. A minor difference is that the group of CI-users with right-ear stimulation required a longer duration to return to baseline activity.*

**CI artifacts in ICA**

**
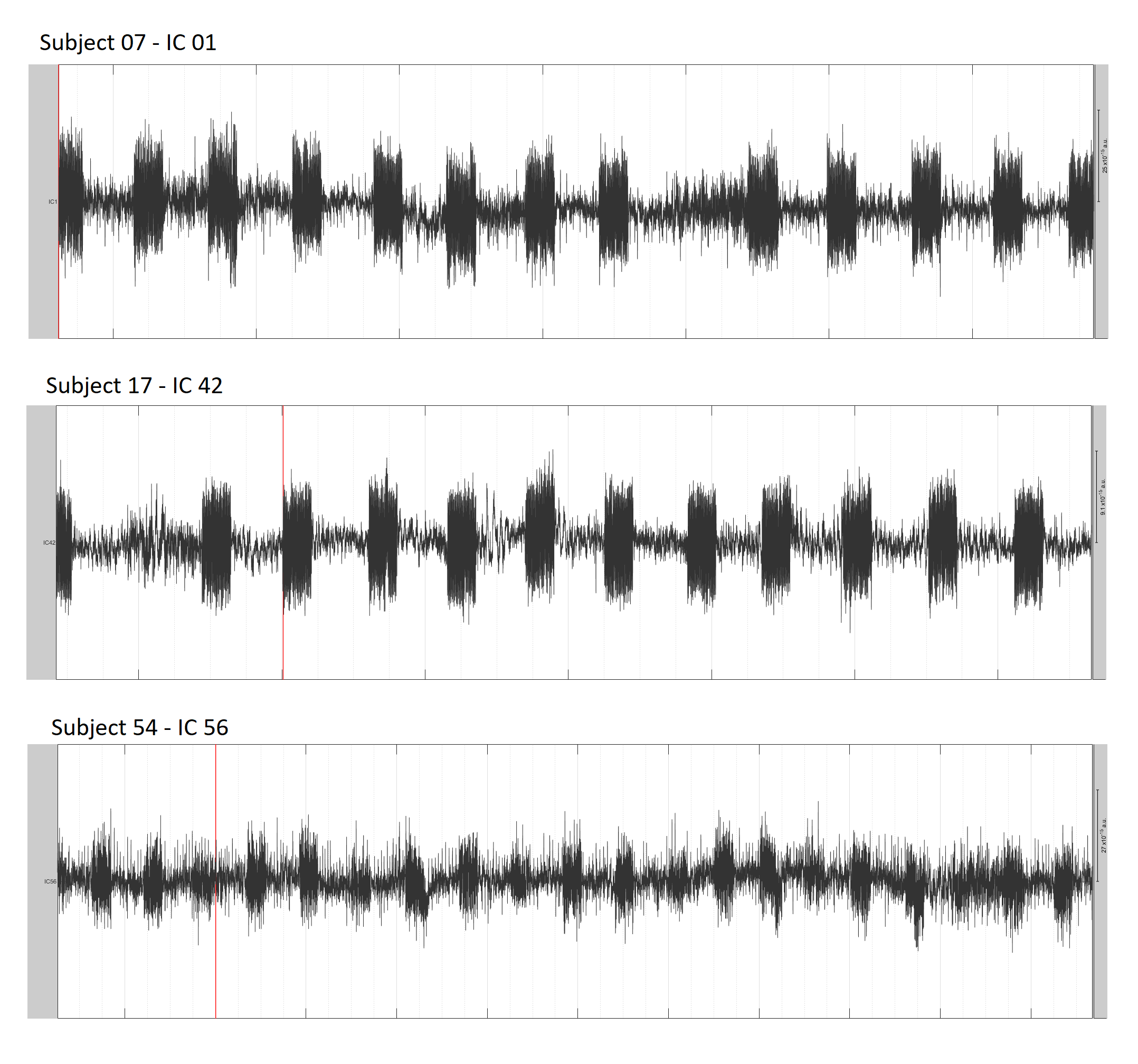
**

***Supplementary Fig. 2: Representative samples of independent components (ICs) resembling CI-related artifacts.*** *The CI-related artifacts appear in the independent component analysis (ICA) as stimulus time-locked signals, whose durations closely match the stimulus durations.*
